# Cancer somatic mutations cluster in a subset of regulatory sites predicted from the ENCODE data

**DOI:** 10.1186/s12943-016-0560-0

**Published:** 2016-11-25

**Authors:** Nisar A. Shar, M. S. Vijayabaskar, David R. Westhead

**Affiliations:** 1 School of Molecular and Cellular Biology, Garstang Building, University of Leeds, Leeds, LS2 9JT UK; 2 Department of Biomedical Engineering, NED University of Engineering & Technology, University Road, Karachi, 75270 Pakistan

**Keywords:** Cancer mutations, Cis regulation, Gene regulation, Modelling, Regulatory regions

## Abstract

**Background:**

Transcriptional regulation of gene expression is essential for cellular differentiation and function, and defects in the process are associated with cancer. The ENCODE project has mapped potential regulatory sites across the complete genome in many cell types, and these regions have been shown to harbour many of the somatic mutations that occur in cancer cells, suggesting that their effects may drive cancer initiation and development. The ENCODE data suggests a very large number of regulatory sites, and methods are needed to identify those that are most relevant and to connect them to the genes that they control.

**Methods:**

Predictive models of gene expression were developed by integrating the ENCODE data for regulation, including transcription factor binding and DNase1 hypersensitivity, with RNA-seq data for gene expression. A penalized regression method was used to identify the most predictive potential regulatory sites for each transcript. Known cancer somatic mutations from the COSMIC database were mapped to potential regulatory sites, and we examined differences in the mapping frequencies associated with sites chosen in regulatory models and other (rejected) sites. The effects of potential confounders, for example replication timing, were considered.

**Results:**

Cancer somatic mutations preferentially occupy those regulatory regions chosen in our models as most predictive of gene expression.

**Conclusion:**

Our methods have identified a significantly reduced set of regulatory sites that are enriched in cancer somatic mutations and are more predictive of gene expression. This has significance for the mechanistic interpretation of cancer mutations, and the understanding of genetic regulation.

**Electronic supplementary material:**

The online version of this article (doi:10.1186/s12943-016-0560-0) contains supplementary material, which is available to authorized users.

## Background

The majority of work on the somatic mutations that are found in cancer cell genomes has focussed on the analysis of protein coding exons. These regions have clear functional significance, and because they represent only a very small fraction of the genome are more amenable to systematic experimental investigation (e.g. in whole exome sequencing studies). Analysis of these data, taking account of the relationship between mutational frequencies and variables such as replication timing and gene expression, has allowed the identification of recurrently mutated regions and protein coding genes that when mutated are likely to be oncogenic drivers [1].

The role of aberrant genetic regulatory processes in the initiation and progression of cancer, for example the constituent activation of transcription factors driven by chromosomal re-arrangements [2], has been appreciated for many years. More recently, the discovery of point mutations in the TERT gene promoter that occur in large percentages of cases in some cancer types and are strongly linked to gene expression changes [3, 4], along with developments in whole genome sequencing, have focussed the field on mutations that occur in potential regulatory elements within the genome. It has been shown that regulatory regions harbour significant numbers of the somatic mutations that have been observed in cancer cell genomes [5], and this work has also provided some evidence of positive selection for mutations in these regions, suggesting that regulatory mutations may be important in promoting survival and reproduction of cancer cells in the host. Other related work has examined recurrently mutated regulatory elements [6] and discovered regions potentially regulating genes with known involvement in cancer. Further, a method for the discovery of mutations that are strongly linked to expression levels of nearby genes in cancer samples has been developed [7]. However, given the complexity of genetic regulation in eukaryotic cells, it is likely that current work reveals only a fraction of the regulatory aberrations driving cancer, and there is a clear need for new methods that will reveal different insights.

New technologies for DNA sequencing have revolutionised our ability to map regulatory regions of the genome. For example, the ENCODE project [8] has mapped gene expression, transcription factor binding to DNA and other relevant variables such as DNaseI hypersensitivity and chromatin modifications on a whole genome scale in many laboratory cell lines, and more recent studies have examined the regulation of cellular differentiation [9, 10]. These studies and others have led to the development of databases, for example RegulomeDB [11], and these provide a rich source of information on potential regulatory elements. However, genetic regulation operates at multiple levels, and despite the volume of data now available it remains an unmet challenge to convert this data into more detailed mechanistic understanding of the regulation of individual genes. A large number of candidate regulatory elements are identified in the genome by these technologies, and the possibility that genes are regulated by elements that are relatively distant in the genome makes the process of assigning regulatory elements to genes very difficult. Nevertheless, these large data sets allow the development of correlative models whereby candidate regulatory elements may be identified, and used to develop regulatory networks linking them to the genes they control [12]. Similar work has used logistic regression [13], and Thurman and co-workers [14] introduced models that link DNaseI hypersensitivity data in promoter and distal sites to identify regulatory regions. While these methods are clearly useful, independent experimental knowledge of the links between genes and their regulatory regions is presently too limited for effective method comparison and validation.

A useful alternative view of the utility of correlative models of genetic regulation is to examine them in the context of relevant independent biological data, such as the somatic mutations observed in cancer genomes. Here we introduce our own model of genetic regulation based on ENCODE and examine the mapping of cancer mutations from the COSMIC [15] database to the regulatory regions it identifies. This integration of two large public sources of biological information through modelling, has the potential to improve our understanding both of genetic regulation and cancer.

## Results

**Fig. 1:**
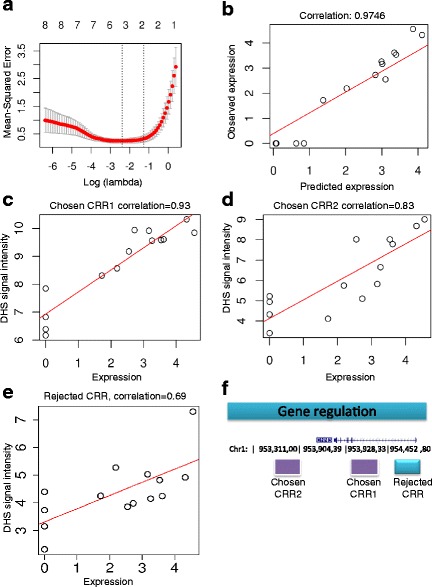
Building an expression model for CNN3 (ENST00000370206.4). **a** shows the mean squared error against the log (λ) LASSO penalty parameter with numbers above the graph indicating the number of predictive variables (non-zero coefficients) in the corresponding LASSO model. Dotted lines show possible choices of λ at minimum mean-squared error (λ_min_) and more conservatively at that value plus 1 standard error. This identifies models with 2 predictive variables as optimal. **b** shows the correlation between observed expression and predicted expression from the model. **c** and **d** show the correlation of DNaseI signal intensities and expression for the two candidate regulatory elements (CRRs) chosen by the LASSO method. **e** shows the correlation between DNaseI signal intensities and expression for an example rejected CRR. **f** shows the genomic location of the two chosen CRRs and one example rejected CRR

Figure 1 illustrates the process of building a simple correlative model of gene expression for a single transcript. As described in the Methods, candidate regulatory regions (CRRs) were identified as the union of all sites of transcription factor binding and the top 25% of DNase1 hypersensitive sites in all the ENCODE cell types considered. Each transcript was considered to be potentially regulated by any CRR within 100 kB [16] of the transcription start site (TSS), in this case 72 CRRs. Although genes can be regulated by enhancers up to 1 MB from the TSS, the figure of 100kB was chosen to encompass most regulatory elements, for example those of the leukaemia related oncogene Lmo2 [17]. The aim of our model was to predict the expression level of the gene in each of the cell types, as measured by RNA-seq experiments, from signal intensities in DNase1 hypersensitivity data, which we use as a crude measure of activity (e.g. transcription factor binding) at the CRR concerned.

Given the large number of CRRs relative to the number of cell types in which gene expression was measured, we adopted a penalised regression approach (LASSO) to identify a small set containing just those elements with the strongest relationships to gene expression. Analysis of the LASSO data indicated that the best supported models were based on just two candidate regulatory elements per transcript. We subsequently refer to these elements as the ‘chosen’ CRRs, and the remaining elements as ‘rejected’ CRRs. In the case of the transcript in Fig. 1 a convincing model was constructed, showing a (Pearson) correlation of observed to predicted expression values of 0.97 (Fig. 1b). We further assessed the statistical significance of this model using a randomisation approach, resulting in a p value of 0.0016 (see Statistical significance of models in Methods). Figure 1c and d show the correlation of DNaseI signal intensities and expression for the two CRRs chosen by the LASSO method, and Fig. 1e shows a example rejected CRR. The genomic location of the CRRs is shown in Fig. 1f.

**Fig. 2:**
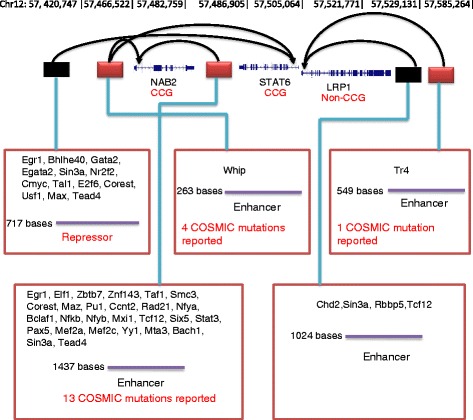
The chosen CRRs for NAB2 (ENST00000342556.5), STAT6 (ENST00000300134.2) and LRP1 (ENST00000243077.2). *Black* arrows link CRRs to the transcripts for which they were chosen in expression models; note that one CRR was chosen for both STAT6 and NAB2. Details of the chosen CRRs are given *red* boxes, including the bound transcription factors, sizes of the CRRs and mutations mapped from the COSMIC database. CRRs are labelled as enhancers if they show positive correlation with expression and repressors if they show negative correlation. The chosen CRRs are marked as red boxes if there is at least one reported mutation in them, and black otherwise

We next investigated the possibility of a large-scale model building exercise for all genes/transcripts, and also in a restricted set of 533 cancer census genes from COSMIC [18]. We focussed on transcripts from GENCODE v7, and restricted the study to transcripts expressed in at least 7 cell types, which were more suitable for our regression based modelling techniques. Thus our study focused on genes expressed in a wider range of cell types, and we call these ‘globally expressed’ genes. The relevant statistics of model building are shown in Table 1, and a list of all chosen CRRs along with their target genes is included in Additional file 1: Table S1. Models were successfully built for approximately 9000 genes (16000 transcripts), and 290 genes (650 transcripts) from the cancer set. It should be noted that the scale of this model building exercise leads, after correction for multiple testing, to a significant false discovery rate. While any individual model should be considered carefully in this light, we treated the exercise as a means to the identification of a single set of CRRs covering a substantial proportion of the transcriptome that lead to the best supported models of gene expression (the chosen set), and a complement set of rejected CRRs with weaker relationships to gene expression. It should be noted that some elements were chosen for more than one transcript, and this is illustrated in Fig. 2, which also highlights the transcription factors known to bind in each CRR. As an illustration of the results for more genes, in Additional file 2: Figures S1 and S2 we include four examples (*WNT5A*, *ID1*, *LIMS1* and *TEAD3*) where predicted CRRs coincide with regulatory elements that are already known [19].

**Table 1:** Statistics of model building

	All transcripts	Cancer set transcripts
Number of models attempted	17963 transcripts from 9209 genes	731 transcripts (from 304 genes)
Number of models built	16134 (8670 genes)	654 (292 genes)
Average r,*r* ^2^	0.710, 0.519	0.718, 0.530
Range *r* ^2^	0.004–0.99	0.048–0.925
Total candidate elements	678020 (mean 42/transcript)	28844 (mean 44/transcript)
Chosen elements	25045 (2/transcript)	1140 (2/transcript)
Elements chosen for 1 transcript	20025	999
Elements chosen for >1 transcript	5020	141

The COSMIC database [15] is a high-quality compilation of somatic mutations that have been observed in cancer cells. Mutations were downloaded from this database (a total of 2.3 million mutations) and mapped to the CRRs, as illustrated in Fig. 2. Overall 8% of these mutations mapped to CRRs identified with the transcript set defined above, and 14% of transcripts mapped to at least one mutated CRR. Table 2 gives statistics showing how these mutations are partitioned between chosen and rejected CRRs from the modelling exercise. This shows that a significantly higher proportion of chosen CRRs are mutated at least once compared rejected CRRs, and that chosen CRRs harbour around 1.5 times more mutations than rejected CRRs. This applies equally to all genes and to the cancer related subset. Within the all genes set all comparisons are highly statistically significant, while the smaller cancer genes set shows the same trends but with reduced levels of statistical significance. When limiting the analysis to CRRs only from higher quality expression models (confident models with *r* > 0.7 and highly confident models with *r* > 0.8) the effect size increases: mutations are enriched in chosen CRRs by a factor of 1.45 (=1.35/0.93) in all models and this rises to 1.81 in CRRs for highly confident models.

**Table 2:** Mapping of somatic mutations from COSMIC to candidate regulatory regions (CRRs)

Title	All		Cancer census genes	
	Chosen CRRs	Rejected CRRs	Chosen CRRs	Rejected CRRs
Total number of CRRs	25045	158560	1140	7429
CRRs mutated at least once	3535 (14.11%)^1^	16241 (10.24%)^1^	160 (14.03%)^2^	703 (9.46%)^2^
Mean mutations/CRR	1.35^3^	0.93^3^	1.51^4^	0.97^4^
Mean mutations/CRR (models with *r* > 0.7)	1.40^3^	0.88^3^	1.63^5^	0.95^5^
Mean mutations/CRR (models with *r* > 0.8)	1.50^3^	0.83^3^	1.55	0.92

^1^Proportion mutated in chosen set greater than in rejected set, *p* < 10^−15^ (Chi-squared and Fisher test)

^2^Proportion mutated in chosen set greater than in rejected set, *p* < 10^−5^ (Chi-squared and Fisher test)

^3^Mean mutations in chosen set greater than in rejected set, *p* < 10^−23^ (two sample t test), *p* < 10^−8^ (Wilcoxon test)

^4^Mean mutations in chosen set greater than in rejected set, *p* < 0.05 (two sample t test and Wilcoxon test)

^5^Mean mutations in chosen set greater than in rejected set, *p* < 0.05 (two sample t test), *p* = 0.06 (Wilcoxon test)

It is known that DNA mutation frequencies are heterogeneous [1] over the genome, and are related to variables such as replication timing and GC content. Equally, in the context of this analysis, average mutation frequencies within CRRs might be expected to be affected by the length of the CRR and possibly the proximity to a transcription start site (TSS). We took two different approaches to investigate whether these effects could have biased the statistical considerations above. First we repeated the significance tests on the mean number of mutations per CRR, this time not using the entire set of rejected CRRs but by randomly choosing a set of equal size to the chosen set matched according to the variable concerned (e.g. matching each chosen set member with a rejected member falling in the same GC content bin). In the case of all variables (replication timing, GC content, length of CRR and proximity to a TSS) the effects reported above remained significant, albeit with reduced levels of significance reflecting the reduction in size of the rejected set. Second, to model all these potential effects simultaneously we built generalised linear models for the counts of mutations in CRRs. We found the counts to be over-dispersed with respect to a Poisson distribution assumption, and modelled this with an additional dispersion parameter (see Methods). The effect size for an indicator variable showing whether a CRR was chosen or rejected was 0.46+/−0.02 (*p* < 2 × 10^−16^,Wald test), revealing a highly significant effect on the (log) expected mutation counts consistent in size with observed differences in average mutation counts from Table 2.

Finally, within the chosen set of CRRs we tested for differences in the average number of mutations in different types of CRR. Chosen CRRs may be positively or negatively correlated with expression of the associated gene, and hence tentatively identified with enhancing or repressing mechanisms. Of our chosen CRRs 32% showed negative correlations with expression, but there was no significant difference in mutation rates between these two types of CRR, whether considering all models or just those from cancer associated genes. On the other hand dividing CRRs into proximal or distal according to distance from the associated transcription start site (distal > 10 kB, proximal < 10 kB) showed a significant tendency for proximal CRRs to be mutated to higher levels, as shown in Table 3 and previously reported [6]. This effect seems to be more pronounced in elements identified with cancer associated genes.

**Table 3:** Mapping of mutations to chosen CRRs proximal and distal to the transcription start site

	Proximal (<10kB from TSS)	Distal (>10kB from TSS)
Mean mutations/CRR (all models)	2.30^1^	1.25^1^
Mean mutations/CRR (cancer related transcripts)	3.40^2^	0.99^2^

^1^Mean greater in proximal set, *p* < 10^−39^ (*t*-test), *p* < 10^−17^ (Wilcoxon)

^1^Mean greater in proximal set, *p* < 10^−6^ (*t*-test), *p* < 0.05 (Wilcoxon)

## Discussion

The recent revolution in DNA sequencing speed has allowed us to map multiple variables relevant to genetic regulation at genome-scale and sequence the genomes of many individual cancers. The work reported here is relevant to two important problems that arise from this data: the first is to move from a descriptive understanding of potential regulatory regions to a mechanistic understanding of the regulation of individual genes, and the second to understand which somatic mutations in cancer cells drive the process of cancer progression and to identify underlying mechanisms.

In respect of the problem of understanding genetic regulation, the genome scale data sets we have presently still represent relatively little data for each individual gene or transcript. The complexity of regulation in eukaryotic cells, involving the interactions of transcription factors and chromatin modifiers as well as miRNAs and lncRNAs, and the potential involvement of DNA regions (enhancers) distal to the transcript, mean that our present levels of mechanistic insight are limited. Based on the large scale data we have, the best that is possible is the building of simple correlative models, which aim to identify just those regions of the genome that seem most strongly influential on gene expression. As we have already commented, even this is subject to a significant false discovery rate when attempted at genome-scale. Nevertheless, changes to genetic regulation are an important feature of cancer, and the results reported here show that a set of candidate regulatory regions derived from simple correlative models preferentially harbour cancer somatic mutations, suggesting that these regions are of functional significance in genetic regulation.

## Conclusions

It is now recognised that mutations affecting regulatory regions are potentially as important in cancer progression as mutations in protein coding regions or those that directly alter functional RNA molecules. Here we have shown that somatic mutations that are found in cancer cells occur preferentially in those potential regulatory regions that are revealed by the ENCODE data to be more likely to be directly involved in the regulation of gene expression levels. This adds to the growing body of work in this area strongly suggesting that cancer progression involves positive selection for mutations with regulatory effects. This work also shows that modelling based on large data compendia like ENCODE can identify genomic regions which are potentially more strongly linked to gene expression, and propose links to the regulated genes. This could lead to more effective definition and prioritisation of mechanistic hypotheses for cancer somatic mutations, which will be accessible to confirmation or refutation with further detailed laboratory investigations.

## Methods

### Data sets and identification of candidate cis regulatory regions

Data sets were downloaded from ENCODE [8] for human genome version hg19 as shown in Table 4. Candidate Regulatory Regions (CRRs) were defined as all transcription factor binding sites (TFBS) found in the five cell types for which ChIP-seq data for transcription factors was available, plus the highest scoring 25% of DNaseI hypersensitive (DHS) sites for all 14 cell types, filtered to include only those with the H3K27ac active enhancer mark in at least one cell type. We used DHSs generated by the uniform processing pipeline of the ENCODE Analysis Working Group (AWG) for this study [8], and similarly TFBS were taken from the ENCODE standard data processing pipeline [8].

**Table 4:** ENCODE data sets used

S.No	Cell	ChIP-seq^a^(TFs)	DNaseI-seq	RNA-seq (FPKM)	ChIP-seq (H3K27ac)
1	K562	100	*√*	*√*	*√*
2	Gm12878	73	*√*	*√*	*√*
3	Hepg2	57	*√*	*√*	*√*
4	Helas3	54	*√*	*√*	*√*
5	H1hesc	47	*√*	*√*	*√*
6	A549		*√*	*√*	
7	Ag04450		*√*	*√*	
8	Bj		*√*	*√*	
9	Hsmm		*√*	*√*	
10	Huvec		*√*	*√*	
11	Mcf7		*√*	*√*	
12	Nhek		*√*	*√*	
13	Nhlf		*√*	*√*	
14	Sknshra		*√*	*√*	

^a^Total number of transcription factor ChIP-seq datasets considered, note that data sets of CTCF, CTCFL and RNA polymerase II were not used

The DHS and TFBS were merged if they overlapped by at least 1 base pair using bedtools [20] and the resulting merged regions were considered as the full set of candidate regulatory regions (CRRs) for further analysis. DNaseI-seq signal intensities for each CRR in the 14 cell types (Table 4) were computed from the uniformly processed and normalised signal tracks using bwtool [21].

**Fig. 3:**
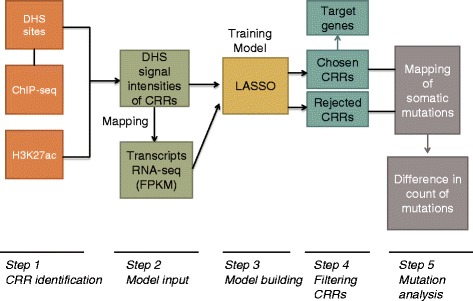
Graphical summary of the methodology employed

RNA-seq (whole-cell polyA+) transcript quantifications were downloaded from the ENCODE DCC portal of UCSC genome browser [22]. The expression for any transcript whose coordinates are defined by GENCODE (version 7) [23] is the average FPKM (Fragments Per Kilobase of transcript per Million sequenced reads) [24] of all the replicates, and they were filtered for IDR (Irreproducible Discovery Rate) < = 0.1. Further, only transcripts that were expressed (FPKM > =1) in at least 7 of the cell types defined in Table 4 were considered for all our analysis given below (such data is more suitable for our regression based modelling scheme). Our methodology is illustrated graphically in Fig. 3.

### Model

For applicability in the largest number of cell types, we based our model on DHS data and assumed a simple linear relationship between transcript expression (log (FPKM) values) and (log (signal intensity)) from the DNaseI data in each CRR.

$$ y={k}_0+{\displaystyle {\sum}_{i=1}^n{k}_i{x}_i} $$

Here *y* is the expression value of the transcript, *x*_*i*_ the DNaseI signal intensity in the *i* ^*th*^ CRR for that transcript and *n* is the number of CRRs within 100 kb of the transcription start site.

Since *n* is typically greater than the number of cell types for which data were available, model fitting demanded a penalised approach to limit the number of non-zero *k*_*i*_ coefficients. We chose LASSO regression implemented in the R glmnet package [25], which represents a least squares/maximum likelihood fit penalised with a term *λ*∑_*i* = 1_^*n*^|*k*_*i*_|. We investigated a number of different ways of determining appropriate values for the penalty scaling parameter *λ,* using a selection of example genes, and eventually chose conservatively so that two non-zero *k*_*i*_ parameters were determined for each model. As shown in Fig. 1, this is consistent with the glmnet package recommendation for choosing *λ*, as either *λ*_*min*_ (minimum mean square error) or this value plus one standard error. CRRs are subsequently referred to as ‘chosen CRRs’ if they appear with a non-zero coefficient in a LASSO model for at least one transcript, and rejected CRRs if they were considered in the analysis for any model but never associated with a non-zero coefficient. In the supplementary material we have included an input data file (Additional file 3) and R code (Additional file 4) to illustrate how the method can be implemented.

### Statistical significance of models

The quality of the models was assessed through the (Pearson) correlation of predicted and observed gene expression values, using a leave-one-out cross validation scheme. To further assess statistical significance we generated models from randomly permuted data: we fixed the DHS data and generated 50000 random permutations of the gene expression values per transcript, calculating the empirical probability of obtaining a model from the random data showing a correlation at least as high as that for the model from the real data (using the same value of *λ* in each case). Since randomization is computationally expensive, we considered 12 models: 4 transcripts where the real model showed high correlation of predicted and actual expression (~0.9), 4 with moderate correlations (~0.5) and 4 where LASSO failed to find models. We found that the distribution of random model correlations was remarkably similar in all these cases and therefore used the distribution from these combined randomizations to generate *p* values for all models. When studying the generation of models for multiple genes we chose to control the false positive rate using the Benjamini-Hochberg method.

### Mapping cancer mutations to regulatory regions

Somatic cancer mutations were derived from the COSMIC database [15] v76 (Catalogue of somatic mutations in cancer). 2.3 million somatic mutations were retrieved and mapped to the CRRs defined above. Duplicate/recurrent mutations were eliminated so only one mutation was considered at each genomic location.

### Statistical significance of differences in mutation counts

The statistical significances of differences in the counts of somatic mutations observed in chosen and rejected CRRs were tested in several ways. Differences in the average number of mutations per CRR were tested with two-sample t-tests, and also equivalent non-parametric Wilcoxon tests to account for possible non-normality. To account for other possible effects that might bias these considerations we also repeated these tests after first balancing the chosen and rejected sets to have the same distribution of any potential confounding variable. This was achieved by sampling the rejected set of CRRs randomly to match the distribution of a variable in the chosen set, which was enabled by the significantly larger size of the rejected set. The variables considered were replication timing, base pair composition, length of the CRRs and distance of the CRR to the transcription start site (TSS). Replication timing and GC content data was downloaded from the UCSC website: the wavelet-smoothed signal of replication timing [26] for 9 cell types was obtained and we used the average signal. In each case data was binned in 4 equal bins and the process required a chosen CRR to be matched by a rejected CRR from the same bin.

As an alternative test of statistical significance which enabled us to model all potential effects on mutation counts together, we built generalised linear models using the glm function in R. Mutation counts were modelled as a function of length of CRR, replication timing, GC content, shortest distance to a TSS and an indicator variable for chosen/rejected CRRs. A log link function was used, first under the assumption of a Poisson distribution for the counts and then in cases of over-dispersion using the quasipoisson option in glm, which fits a dispersion parameter which is otherwise fixed at unity. The statistical significances of the effects of each variable were assessed from the standard Wald test statistics produced by glm.

### Cancer census genes

A set of 533 cancer consensus genes were retrieved from the COSMIC database of which 292 entered our analysis (the others did not meet our modelling criterion of expressing in at least 7 cell types). These were analysed as a separate subset to investigate any possible specific effects for genes known to be directly involved in cancer.

## Additional files

Additional file 1: Identified regulatory sites containing somatic mutations. (XLSX 2006 kb)
Additional file 2: Examples of four genes, where predicted CRRs coincide with the regulatory elements that are already known. (PPTX 63 kb)
Additional file 3: Example of input data fi le for the predictive model. (TXT 14 kb)
Additional file 4: R code for LASSO model building. (R 1 kb)

## Abbreviations

ChIP: Chromatin immunoprecipitation
COSMIC: Catalogue of somatic mutations in cancer
CRRs: Candidate cis regulatory regions
DHS: DNase I hypersensitive Site
ENCODE: Encyclopedia of DNA elements
FPKM: Fragments per kilobase of transcript per million sequenced reads
Glm: Generalized linear model
LASSO: Least absolute shrinkage and selection operator
TFBS: Transcription factor binding sites
TSS: Transcription start site
