# Supplementary material for: Cancer somatic mutations cluster in a subset of regulatory sites predicted from the ENCODE data
Source: Mol Cancer. 2016 Nov 25;15:76. doi: 10.1186/s12943-016-0560-0 (PMC5123355; doi:10.1186/s12943-016-0560-0)
Supplement: Additional file 2: — Examples of four genes, where predicted CRRs coincide with the regulatory elements that are already known. (PPTX 63 kb) [file 12943_2016_560_MOESM2_ESM.pptx]

## Slide 1
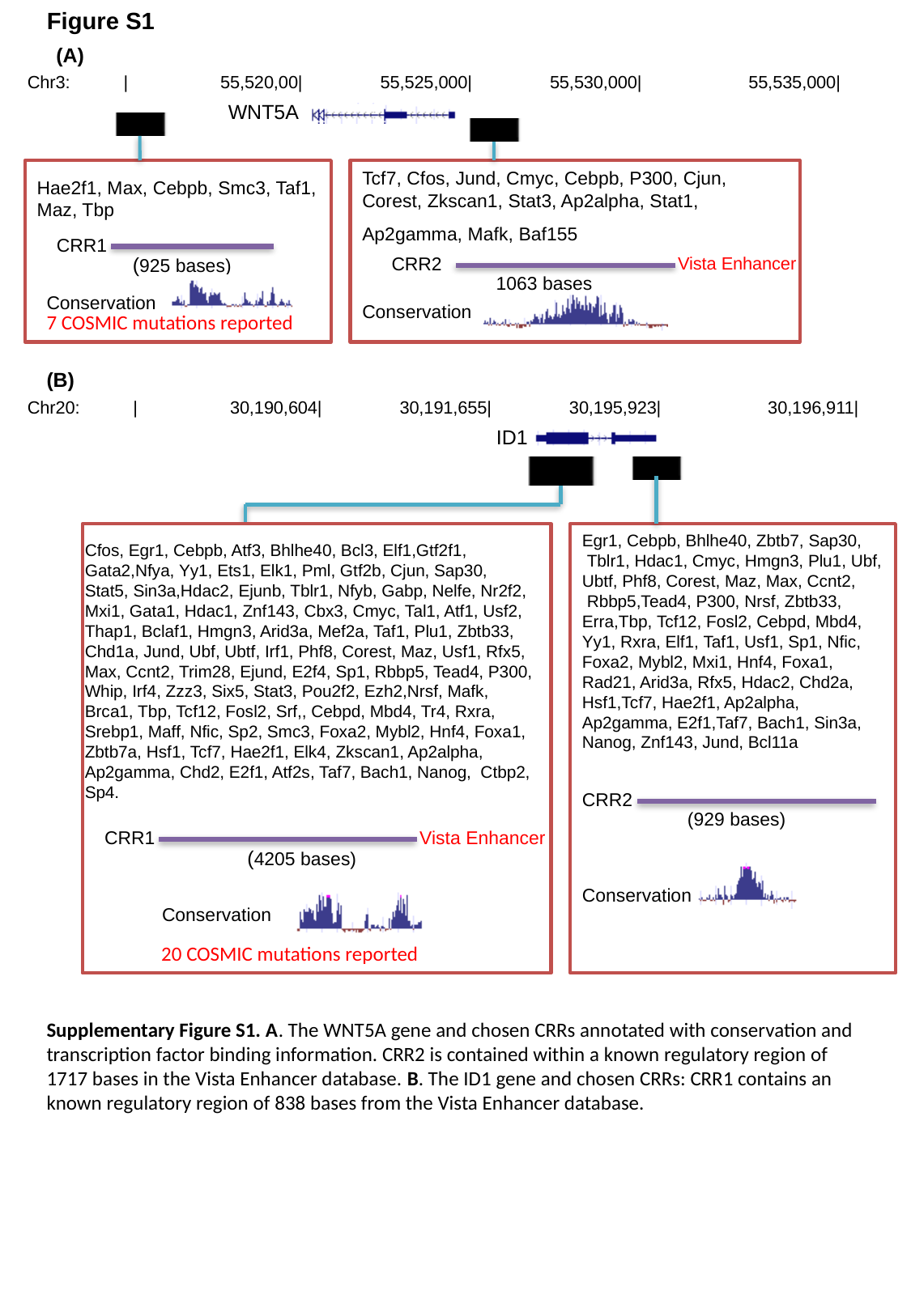

Figure S1
(A)
Chr3: | 55,520,00| 55,525,000| 55,530,000| 55,535,000|
WNT5A
Tcf7, Cfos, Jund, Cmyc, Cebpb, P300, Cjun, Corest, Zkscan1, Stat3, Ap2alpha, Stat1, Ap2gamma, Mafk, Baf155
Hae2f1, Max, Cebpb, Smc3, Taf1, Maz, Tbp
CRR1
CRR2
Vista Enhancer
(925 bases)
1063 bases
Conservation
Conservation
7 COSMIC mutations reported
(B)
Chr20: | 30,190,604| 30,191,655| 30,195,923| 30,196,911|
ID1
Egr1, Cebpb, Bhlhe40, Zbtb7, Sap30,
 Tblr1, Hdac1, Cmyc, Hmgn3, Plu1, Ubf,
Ubtf, Phf8, Corest, Maz, Max, Ccnt2,
 Rbbp5,Tead4, P300, Nrsf, Zbtb33, Erra,Tbp, Tcf12, Fosl2, Cebpd, Mbd4, Yy1, Rxra, Elf1, Taf1, Usf1, Sp1, Nfic, Foxa2, Mybl2, Mxi1, Hnf4, Foxa1, Rad21, Arid3a, Rfx5, Hdac2, Chd2a, Hsf1,Tcf7, Hae2f1, Ap2alpha, Ap2gamma, E2f1,Taf7, Bach1, Sin3a, Nanog, Znf143, Jund, Bcl11a
Cfos, Egr1, Cebpb, Atf3, Bhlhe40, Bcl3, Elf1,Gtf2f1, Gata2,Nfya, Yy1, Ets1, Elk1, Pml, Gtf2b, Cjun, Sap30, Stat5, Sin3a,Hdac2, Ejunb, Tblr1, Nfyb, Gabp, Nelfe, Nr2f2, Mxi1, Gata1, Hdac1, Znf143, Cbx3, Cmyc, Tal1, Atf1, Usf2, Thap1, Bclaf1, Hmgn3, Arid3a, Mef2a, Taf1, Plu1, Zbtb33, Chd1a, Jund, Ubf, Ubtf, Irf1, Phf8, Corest, Maz, Usf1, Rfx5, Max, Ccnt2, Trim28, Ejund, E2f4, Sp1, Rbbp5, Tead4, P300, Whip, Irf4, Zzz3, Six5, Stat3, Pou2f2, Ezh2,Nrsf, Mafk, Brca1, Tbp, Tcf12, Fosl2, Srf,, Cebpd, Mbd4, Tr4, Rxra, Srebp1, Maff, Nfic, Sp2, Smc3, Foxa2, Mybl2, Hnf4, Foxa1, Zbtb7a, Hsf1, Tcf7, Hae2f1, Elk4, Zkscan1, Ap2alpha, Ap2gamma, Chd2, E2f1, Atf2s, Taf7, Bach1, Nanog, Ctbp2, Sp4.
CRR2
(929 bases)
CRR1
Vista Enhancer
(4205 bases)
Conservation
Conservation
20 COSMIC mutations reported
Supplementary Figure S1. A. The WNT5A gene and chosen CRRs annotated with conservation and transcription factor binding information. CRR2 is contained within a known regulatory region of 1717 bases in the Vista Enhancer database. B. The ID1 gene and chosen CRRs: CRR1 contains an known regulatory region of 838 bases from the Vista Enhancer database.

## Slide 2
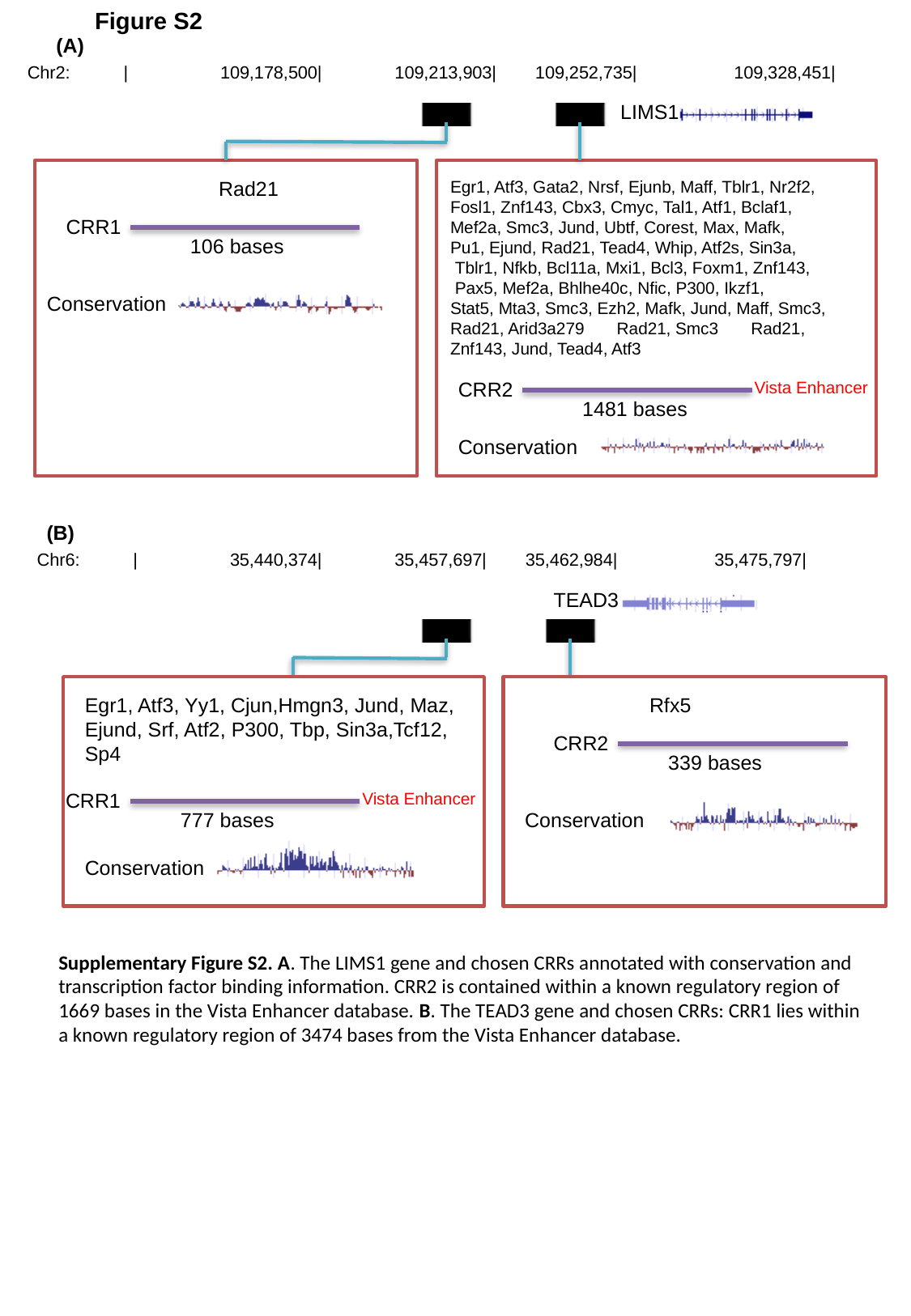

Figure S2
(A)
Chr2: | 109,178,500| 109,213,903| 109,252,735| 109,328,451|
LIMS1
Rad21
Egr1, Atf3, Gata2, Nrsf, Ejunb, Maff, Tblr1, Nr2f2,
Fosl1, Znf143, Cbx3, Cmyc, Tal1, Atf1, Bclaf1,
Mef2a, Smc3, Jund, Ubtf, Corest, Max, Mafk,
Pu1, Ejund, Rad21, Tead4, Whip, Atf2s, Sin3a,
 Tblr1, Nfkb, Bcl11a, Mxi1, Bcl3, Foxm1, Znf143,
 Pax5, Mef2a, Bhlhe40c, Nfic, P300, Ikzf1,
Stat5, Mta3, Smc3, Ezh2, Mafk, Jund, Maff, Smc3,
Rad21, Arid3a279       Rad21, Smc3       Rad21,
Znf143, Jund, Tead4, Atf3
CRR1
106 bases
Conservation
CRR2
Vista Enhancer
1481 bases
Conservation
(B)
Chr6: | 35,440,374| 35,457,697| 35,462,984| 35,475,797|
TEAD3
Egr1, Atf3, Yy1, Cjun,Hmgn3, Jund, Maz, Ejund, Srf, Atf2, P300, Tbp, Sin3a,Tcf12, Sp4
Rfx5
CRR2
339 bases
CRR1
Vista Enhancer
777 bases
Conservation
Conservation
Supplementary Figure S2. A. The LIMS1 gene and chosen CRRs annotated with conservation and transcription factor binding information. CRR2 is contained within a known regulatory region of 1669 bases in the Vista Enhancer database. B. The TEAD3 gene and chosen CRRs: CRR1 lies within a known regulatory region of 3474 bases from the Vista Enhancer database.
